# Fungal Biofilms and Drug Resistance

**DOI:** 10.3201/eid1001.030119

**Published:** 2004-01

**Authors:** Mary Ann Jabra-Rizk, William A. Falkler, Timothy F. Meiller

**Affiliations:** * Dental School, University of Maryland, Baltimore, Maryland, USA

**Keywords:** *Candida albicans*, *Candida dubliniensis*, biofilms, antifungal drug resistance, Echinocandins

## Abstract

*Candida* species, including the novel opportunistic pathogen *Candida dubliniensis*, are now emerging as major agents of nosocomial infections. Many such manifestations of infections associated with the formation of *Candida* biofilms include those occurring on devices such as indwelling intravascular catheters. Fungal biofilm-associated infections are frequently refractory to conventional therapy because of resistance to antimicrobial agents. This resistance could be in part due to the surface-induced upregulation of drug efflux pumps. Biofilm-associated *Candida* show uniform resistance to a wide spectrum of the currently available conventional antifungal agents, which implies that antimicrobial drugs that specifically target biofilm-associated infections are needed. The novel classes of antifungal agents, the lipid formulation of amphotericins, and the echinocandins have demonstrated unique antifungal activity against the resistant *Candida* biofilms, providing a breakthrough in the treatment of life-threatening invasive systemic mycoses. The use of drugs effective in combating biofilm-associated infections could lead to major developments in the treatment of fungal implant infections.

The genus Candida is composed of an extremely heterogeneous group of organisms that grow as yeasts. Most members of the genus also produce a filamentous type of growth (pseudohyphae) (*1*). In addition to pseudohyphae, Candida albicans and C. dubliniensis form true hyphae (germ tubes) and thick-walled cells referred to as chlamydospores, both of which are used by mycology diagnostic laboratories in identifying these species (*1*). Candida species are now emerging as major agents of hospital-acquired infections; they are ranked as the third or fourth most commonly isolated bloodstream pathogens, surpassing gram-negative bacilli in frequency (*2*–*9*). Although C. albicans is the predominant etiologic agent of candidiasis, other Candida species that tend to be less susceptible to the commonly used antifungal drugs such as C. krusei, C. glabrata, C. lusitaniae, and the newest Candida species, C. dubliniensis, have emerged as substantial opportunistic pathogens (*10*). Candida dubliniensis shares with C. albicans many virulence factors, such as germ tube formation, exoenzyme production, and phenotypic switching (*10*). This species, however, unlike C. albicans, has been shown to readily develop stable resistance to fluconazole in vitro and in infected patients, strongly suggesting that C. dubliniensis possesses a readily inducible fluconazole resistance mechanism (*11*–*13*).

Indwelling intravascular catheters represent a risk factor that is associated with nosocomial Candida infections. The devices become colonized by the microorganisms that form a biofilm of cells, the detachment of which can result in septicemia (*2*–*5*,*8*,*9*,*14*,*15*). Most manifestations of candidiasis are in fact associated with the formation of Candida biofilms on surfaces, and this phenotype is associated with infection at both the mucosal and systemic sites (*8*). Superficial Candida infections of prostheses and implanted devices are troublesome and the most frequently encountered. One of the most common is oral denture stomatitis, a Candida infection of the oral mucosa promoted by a close-fitting upper denture present in 65% of edentulous persons (*5*,*8*).

## Microbial Biofilms

Biofilms are universal, complex, interdependent communities of surface-associated microorganisms. The organisms are enclosed in an exopolysaccharide matrix occurring on any surface, particularly aquatic and industrial water systems as well as medical devices. As such, biofilms are highly relevant for public health (*4*,*7*,*15*–*18*). Most microorganisms grow in structured biofilms rather than individually in suspensions and while in this environment may display altered phenotypes (*2*). Biofilms can be composed of a population that developed from a single species or a community derived from multiple microbial species (*14*,*17*). Speculations about the ecologic advantages of forming a biofilm include protection from the environment, nutrient availability, metabolic cooperation, and acquisition of new genetic traits (*3*,*17*). Biofilms are notoriously difficult to eliminate and are a source of many recalcitrant infections (*15*,*16*). A variety of microbial infections are caused by biofilms ranging from the common such as urinary tract infections, catheter infections, child middle-ear infections, and dental plaque to more threatening infections, such as endocarditis and infections of heart valves (*16*,*19*). Immunocompromised patients such as those with cancer or HIV infection are often the most susceptible.

**Figure 1:**
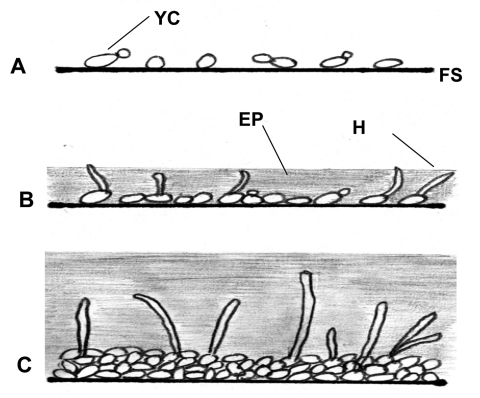
Illustration of biofilm development in Candida albicans and C. dubliniensis; A, early 0–11 h; B, intermediate 12–30 h; C, mature 38–72 h; FS, flat surface; YC, yeast cell; H, hyphae; EP, exopolymeric matrix.

**Figure 2:**
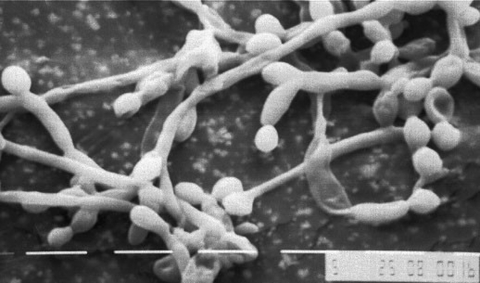
Typical field found in scanning electron micrograph of biofilm formed by Candida albicans on intravascular disc prepared from catheter material

Although bacterial biofilms and their role in disease have been investigated in detail over a number of years, much less is known about fungal biofilms (*2*,*3*,*8*,*9*). Regarding oral or pharyngeal infections, to colonize and infect the oral environment, yeast cells must first adhere to host cells and tissues or prosthetic materials within the oral cavity or must coaggregate with other oral microorganisms (*8*,*20*,*21*). C. albicans biofilm formation has been shown in our laboratory and others to proceed in three distinct developmental phases: early (0–11 h), intermediate (12–30 h), and mature (38–72 h) (*5*) (Figure 1). The detailed structure of a mature C. albicans biofilm produced in vitro after 48-hour incubation has been shown to consist of a dense network of yeasts, hyphae, and pseudohypha (Figure 2). This mixture of yeasts, hyphae, and matrix material is not seen when the organism is grown in liquid culture or on an agar surface, which suggests that morphogenesis is triggered when an organism contacts a surface and that the basal cell layer may have an important role in anchoring the biofilm to the surface (*2*,*3*,*5*,*8*). In addition, bacteria are often found with Candida species in biofilms in vivo, indicating that extensive interspecies interactions probably occur (*2*,*3*,*14*,*18*,*20*).

Candida biofilms share several properties with bacterial biofilms. The two consequences of biofilm growth with profound clinical implications are the markedly enhanced resistance to antimicrobial agents and protection from host defenses, the main reasons why biofilm-associated infections are frequently refractory to conventional therapy (*2*,*4*,*5*,*7*–*9*,*16*,*18*,*22*,*23*). Recently, studies showed that C. dubliniensis has the ability to adhere to and form biofilms with structural heterogeneity and typical microcolony and water channel architecture similar to what has been described for bacterial biofilms and C. albicans biofilms (*7*,*8*). In addition, resistance of C. dubliniensis to fluconazole, as well as increased resistance to clinically applied amphotericin B (*8*,*12*,*13*,*23*,*24*), was demonstrated in biofilms.

## Antifungal-Drug Resistance

Antifungal drug resistance is quickly becoming a major problem in the expanding population of immunocompromised persons. It has resulted in a drastic increase in the incidence of opportunistic and systemic fungal infections. Clinical resistance is defined as persistence or progression of an infection despite appropriate antimicrobial therapy. Resistance is considered primary when an organism is resistant to the drug before exposure, whereas secondary resistance is that which develops in response to exposure to the drug (*25*). This latter mechanism of resistance accounts for the emergence of resistance to azoles seen over the last few years. Azole antifungal agents have become important in the treatment of mucosal candidiasis in HIV patients. Specifically, fluconazole is considered the drug of choice for the most common HIV-associated opportunistic infections in the oral cavity (*26*). Increased use of the azoles, coupled with the fact that they are fungistatic drugs, has likely resulted in the emergence of resistance to azoles.

Major genes that contribute to drug resistance are those coding for multidrug efflux pumps, the upregulation of which can result in a multidrug-resistant phenotype (*2*,*5*,*9*,*26*,*27*). C. albicans and C. dubliniensis possess two different types of efflux pumps: adenosine triposphate–binding cassette (ABC) transporters encoded by the CDR genes (CDR1 and CDR2) and major facilitators encoded by the MDR genes (*2*,*12*,*26*–*28*). Genes for both types of efflux pumps have been recently demonstrated to be upregulated during biofilm formation and development (*2*,*5*,*9*). The ABC transporters CDR1 and CDR2 in C. albicans and C. dubliniensis constitute a multigene family with a demonstrated role in resistance (*5*,*9*,*12*). The MDR1 gene encodes a major facilitator, the overexpression of which leads exclusively to fluconazole resistance (*5*,*9*,*12*).

## Antimicrobial-Drug Resistance

Microbial biofilms not only serve as a nidus for disease but also are often associated with high-level antimicrobial resistance, a consistent phenomenon that may explain the persistence of many infections in the face of appropriate antimicrobial therapy (*15*,*29*). A study by Ramage et al. (*9*) analyzed the expression of C. albicans MDR1, CDR1, and CDR2 genes during both planktonic and biofilm modes of growth. Yeast biofilms were formed in the wells of microtiter plates by pipetting standardized cell suspension of freshly grown and washed yeast cells into wells of microtiter plates and incubating at 37°C (*9*). After biofilm formation, the medium was aspirated and nonadherent cells were removed by thoroughly washing the biofilm. Antifungal susceptibility testing was performed by adding antifungal solution to the biofilms in serially diluted concentrations and incubating for 48 hours at 37ºC. MICs for biofilm cells were determined by using the XTT reduction assay, which semiquantitatively measures the metabolic activity of the cells within the biofilm based on a color change on the reduction of a salt that is reduced by mitochondrial dehydrogenases of metabolically active yeast cells (*9*).

Northern blot analysis from the study showed that mRNA levels for these genes were upregulated when the C. albicans cells were in a sessile mode of growth compared with planktonic cells, with mRNA levels for the MDR1 gene transiently increased in 24-hour biofilms, which indicates that efflux pumps are upregulated in cells within a biofilm, possibly contributing to the observed azole resistance (*9*). However, mutant strains deficient in efflux pumps and hypersusceptible to fluconazole when grown in a planktonic mode retained a resistant phenotype during biofilm growth. This finding demonstrates that drug resistance in biofilms is complex and involves more than one mechanism (*8*).

The mechanisms by which Candida biofilms resist the functions of antifungal agents are therefore poorly understood. Factors that have been considered to be responsible for the increased resistance to antibiotics in bacterial biofilms include restricted penetration of antimicrobials caused by the exopolymeric material (EP) (*14*). Baillie et al. (*4*) analyzed the composition of C. albicans biofilms by isolating EP from catheter tips with adherent biofilm and, after removing the cells in suspension, concentrating and dialyzing the supernatant. The concentrated supernatant was then analyzed for total carbohydrate, phosphorous, protein, glucose, and hexosamine by chemical methods and by high-pressure liquid chromatography. Results of that study showed that the extent of matrix formation in Candida biofilm did not appear to affect the susceptibility of biofilms to five clinically important antifungal agents.

The potential for drug exclusion by the biofilm matrix that may act as a barrier to fluconazole penetration in biofilms of mixed species of Candida and oral bacteria seems to depend on a number of factors; data supporting this mechanism of resistance in bacterial biofilm are strong (*2*,*4*,*7*,*8*,*17*). Growth rate has been considered as an important modulator of drug activity in bacterial biofilms. Biofilms are thought to grow slowly because nutrients are limited, resulting in decreased metabolism of the microorganisms (*2*,*7*,*8*,*16*,*29*). A slow growth rate is frequently associated with the adoption of a different phenotype by microorganisms such as changes in the cell envelope, which in turn affect the susceptibility of the microorganism to antimicrobial agents. In addition, virtually all antimicrobial drugs are more effective in killing rapidly growing cells, and some have an absolute requirement for growth in order to kill (*16*).

Regarding fungal biofilms, however, a study by Chandra et al. (*5*), related to the increase of antifungal resistance during biofilm development, showed that the progression of drug resistance was associated with increase in metabolic activity of the developing biofilm and was not a reflection of slower growth rate, which indicates that drug resistance develops over time, coincident with biofilm maturation. This was the first report correlating the emergence of antifungal drug resistance with the development of biofilm (*4*).

Since the drug resistance in C. albicans biofilms cannot be attributed solely to matrix exclusion or slow growth rate, contact-induced gene expression for acquiring characteristic properties is probably an additional mechanism by which drug resistance is acquired (*4*,*15*). In addition, synthesis of new proteins occurs after C. albicans attaches to surfaces, which suggests that drug resistance might also arise as a consequence of specific surface-induced gene expression (*4*). Quantitative analysis of planktonic EP in comparison to C. albicans biofilm EP showed that glucose was more abundant in biofilm EP than planktonic EP, also suggesting that C. albicans might produce biofilm-specific EP by differentially regulating genes encoding enzymes involved in carbohydrate synthesis (*4*,*5*). In addition, the expression profile of C. albicans genes belonging to the ALS family, which encode proteins implicated in adhesion of C. albicans to host surfaces, was investigated. Northern blot analysis of total RNA from planktonic and biofilm-grown cells demonstrated that ALS gene expression is differentially regulated between the two growth forms, with additional genes expressed in biofilms (*4*,*5*). These observations provide further evidence for contact-induced gene expression and transcriptional changes that are likely to occur during biofilm formation.

A recently proposed hypothesis on bacterial biofilm drug resistance asserts that most cells in the biofilm may not necessarily be more resistant to killing than planktonic cells. Rather, a few persisters survive and are preserved by the presence of an antimicrobial drug that slows their growth, paradoxically helping persisters to persevere and resist being killed. Thus persisters are ultimately responsible for the high level of biofilm resistance to killing (*8*,*16*,*22*,*29*). The nature of persistence and whether it even applies to fungal biofilms, however, is not clearly understood. The ability to eliminate defective cells that would otherwise drain limited resources may be a substantial adaptive value to a clonal population such as a biofilm community. Cells with serious defects undergo programmed cell death (PCD). Antimicrobial drugs that do not kill cells but cause damage trigger suicide, resulting in death from apoptosis. Persisters could represent cells with disabled PCD as a safety mechanism aimed at preventing suicide when a antimicrobial drug reaches the entire population or when nutrients are limited. Therefore, inhibition of PCD to prevent suicide allows starved cells to develop tolerance to antimicrobial drugs (*16*).

With fungal biofilms serving as a safe reservoir for the release of infecting cells into the oral or other environment, biofilm formation by C. dubliniensis and C. albicans likely represents a key factor in their survival, with important clinical repercussions. Treating life-threatening invasive mycoses with new antifungal agents that are active against biofilms and effective in combating biofilm-associated infections is important. Recently, studies showed some antibiofilm activity with the new lipid formulations of amphotericin B and the two echinocandins (caspofungin and micafungin), a new class of antifungals (*2*,*24*,*29*). These interesting findings could lead to important developments in the treatment of fungal implant infections.

## Class of Antifungal Drugs

The antifungal agents currently available for the treatment of systemic fungal infections are classified by their site of action in fungal cells. The polyene antifungal agents, which include nystatin and amphotericin B, are fungicidal and have the broadest spectrum of antifungal activity of the available agents (*30*,*31*). The polyenes cause the fungal cell to die by intercalating into ergosterol-containing membranes, the major sterol in fungal membrane, to form channels and destroy the proton gradient in the cell with leakage of cytoplasmic content (*30*,*31*). Intravenous amphotericin B has been the drug of choice for invasive fungal infections (*30*). The most serious side effect of amphotericin B therapy is nephrotoxicity. To reduce the nephrotoxicity of conventional amphotericin B, lipid formulations are being used that have comparable antifungal activity but differ in the pharmacologic and toxicologic properties (*24*).

The azoles comprise the second class of antifungal agents and include the imidazoles (clotrimazole, miconazole, and ketoconazole) and the triazoles (fluconazole and itraconazole). The azoles inhibit ergosterol biosynthesis through their interactions with the enzyme lanosterol demethylase, which is responsible for the conversion of lanosterol to ergosterol in the fungal cell membrane, leading to the depletion of ergosterol in the membrane (*30*,*31*). Fluconazole is well tolerated with very low incidence of side effects and is the most effective agent for the treatment of oropharyngeal and vaginal candidiasis, as well as prophylaxis for fungal infections in neutropenic patients undergoing bone marrow transplantation and for oropharyngeal candidiasis in HIV-infected persons (*30*).

5-Flucytosine (5-FC) are nucleoside analogs and constitute the third class of antifungal agents. After its uptake into the fungal cell, 5-FC ultimately leads to the disruption of DNA and protein synthesis of the fungal cell (*30*,*31*). Flucytosine is primarily used in combination with amphotericin B for the treatment of candida endophthalmitis and cryptococcal meningitis (*30*,*31*).

## New Classes of Antifungal Drugs

The echinocandins and their analogs, the pneumocandins, represent the newest class of antifungal drugs (*19*,*29*,*31*–*40*). They inhibit the synthesis of 1,3-β-D-glucan, a fundamental component of the fungal cell wall by the inhibition of 1,3 β-D-glucan synthase, an enzyme complex that forms glucan polymers in the cell wall and is absent in mammalian cells. The inhibition is effective and specific, and brief exposure leads to cell death. The potent antifungal activity of the echinocandins against Candida species was demonstrated by Cuenca-Estrella et al. (*33*) and Quindos et al. (*24*), who evaluated the in vitro activity of LY303306, a semi-synthetic echinocandin B derivative, against 156 clinical isolates of Candida species and 36 C. dubliniensis clinical isolates, respectively. Results showed that LY303366 had potent activity against several Candida species including C. albicans, C. tropicalis, as well as C. glabrata and C. krusei, two species usually considered refractory to azoles. Similarly, 100% of the isolates were susceptible to the new antifungal drugs, indicating that echinocandins may provide new alternatives to fluconazole for treating C. dubliniensis infections (*24*). The excellent in vitro activity of echinocandins demonstrated against fluconazole-resistant Candida species strains indicates that the echinocandins are very promising as novel antifungal agents with important implications for the treatment of infections by these yeasts (*24*,*33*,*34*). Their unique mode of action and their specificity to fungal cell walls result in minimal toxicity to mammalian cells.

## Discussion

By using models of C. albicans biofilms, several studies have shown uniform resistance of the organisms in the biofilm to a wide spectrum of conventional antifungal agents including resistance to the new triazoles (VRC and Ravu), which have been shown to be fungicidal with extended activity against many azole-resistant organisms. Therefore, biofilm-associated infections are difficult to treat, which emphasizes the need to develop antimicrobial drugs that show activity against biofilm-associated organisms and specifically target biofilm-associated infections (*5*,*19*). The novel classes of agents, namely the lipid formulation of amphotericins and the echinocandins, have been shown to have unique activities against the resistant Candida biofilms (*19*,*29*). However, given their large size, that liposomal amphotericin B formulations could penetrate ECM to target the fungal cell wall is somewhat surprising. Their dispersion in phospholipids may in fact facilitate passage through the charged polysaccharide ECM, which may be the mechanism by which these compounds penetrate tissues (*29*). The mechanism of the echinocandins against biofilm cells is still unclear. The echinocandins probably do not exert their antibiofilm effects primarily on the fungal cell wall since only minimal cellular changes have been observed on biofilm-associated Candida cells. One explanation may lie in their potential effect on ECM kinetics, where the inhibition of polysaccharide production by echinocandins could lead to lysis and dissolution of the ECM (*29*). Further studies to determine the exact mode of action of echinocandins on Candida biofilms are warranted.

In conclusion, the amphotericin B lipid formulations and the echinocandins exhibit novel activity against Candida biofilms. The use of these drugs may represent an important step in the treatment of invasive systemic Candida infections by enhancing retention of affected intravascular devices and obviating the need for valve surgery in Candida endocarditis (*2*,*19*,*29*). More importantly, these antifungal drugs may be useful in management of biofilm infections by fungi and may have other clinical applications including those of oral diseases and prostheses rejection.
